# Dynamic monitoring of biomass of rice under different nitrogen treatments using a lightweight UAV with dual image-frame snapshot cameras

**DOI:** 10.1186/s13007-019-0418-8

**Published:** 2019-03-27

**Authors:** Haiyan Cen, Liang Wan, Jiangpeng Zhu, Yijian Li, Xiaoran Li, Yueming Zhu, Haiyong Weng, Weikang Wu, Wenxin Yin, Chi Xu, Yidan Bao, Lei Feng, Jianyao Shou, Yong He

**Affiliations:** 1 College of Biosystems Engineering and Food Science, Zhejiang University, Hangzhou, 310058 People’s Republic of China; 2 Key Laboratory of Spectroscopy Sensing, Ministry of Agriculture and Rural Affairs, Hangzhou, 310058 People’s Republic of China; 3 Zhuji Agricultural Technology Extension Center, Zhuji, 311800 People’s Republic of China

**Keywords:** Unmanned aerial vehicle (UAV), Image-frame snapshot multispectral camera, Data fusion, Aboveground biomass, Crop surface model, Random forest regression

## Abstract

**Background:**

Unmanned aerial vehicle (UAV)-based remote sensing provides a flexible, low-cost, and efficient approach to monitor crop growth status at fine spatial and temporal resolutions, and has a high potential to accelerate breeding process and improve precision field management.

**Method:**

In this study, we discussed the use of lightweight UAV with dual image-frame snapshot cameras to estimate aboveground biomass (AGB) and panicle biomass (PB) of rice at different growth stages with different nitrogen (N) treatments. The spatial–temporal variations in the typical vegetation indices (VIs) and AGB were first investigated, and the accuracy of crop surface model (CSM) extracted from the Red Green Blue (RGB) images at two different stages were also evaluated. Random forest (RF) model for AGB estimation as well as the PB was then developed. Furthermore, variable importance and sensitivity analysis of UAV variables were performed to study the potential of improving model robustness and prediction accuracies.

**Results:**

It was found that the canopy height extracted from the CSM (Hcsm) exhibited a high correlation with the ground-measured canopy height, while it was unsuitable to be independently used for biomass assessment of rice during the entire growth stages. We also observed that several VIs were highly correlated with AGB, and the modified normalized difference spectral index extracted from the multispectral image achieved the highest correlation. RF model with fusing RGB and multispectral image data substantially improved the prediction results of AGB and PB with the prediction of root mean square error (RMSEP) reduced by 8.33–16.00%. The best prediction results for AGB and PB were achieved with the coefficient of determination (r^2^), the RMSEP and relative RMSE (RRMSE) of 0.90, 0.21 kg/m^2^ and 14.05%, and 0.68, 0.10 kg/m^2^ and 12.11%, respectively. In addition, the result confirmed that the sensitivity analysis could simplify the prediction model without reducing the prediction accuracy.

**Conclusion:**

These findings demonstrate the feasibility of applying lightweight UAV with dual image-frame snapshot cameras for rice biomass estimation, and its potential for high throughput analysis of plant growth-related traits in precision agriculture as well as the advanced breeding program.

**Electronic supplementary material:**

The online version of this article (10.1186/s13007-019-0418-8) contains supplementary material, which is available to authorized users.

## Background

Rice (*Oryza sativa*) is one of the most important grain crops worldwide, and it serves as a food staple for more than half of the world’s population [1]. Crop biomass defined as the averaged dry weight per unit area is an important agronomic trait linked to plant genetics, growth rate, and productivity. It is also a key ecological indicator of light use efficiency and carbon stocks in agro-ecosystems [2]. Moreover, biomass can be applied to quantify the grain yield with the harvest index [3]. It is also frequently used to assess crop health status and nutrient supply to support agricultural management practices [4]. Hence, it is necessary to explore advanced and efficient technologies for dynamically monitoring crop biomass during the entire growth stages.

Traditional measurement of biomass mainly relies on the field survey with destructive sampling, which is time-consuming and labor-intensive. Many studies associated with advanced remote sensing methods utilized hand-held instruments (i.e., ASD FieldSpec Pro spectrometer) [5, 6], ground platforms (i.e., manned ground vehicle with laser scanner) [7, 8] and satellite imaging (i.e., Landsat, MODIS, SPOT5, and WorldView-2) [9, 10] for biomass estimation of different crops. However, limited spatial and temporal resolutions, and high cost of obtaining satellite image data, and image quality affected by atmospheric conditions pose great challenges to achieve an accurate estimation of biomass during the whole growth period. Although hand-held devices and ground platforms provide a better spatial resolution and can be used to conduct a field survey as frequently as needed throughout the crop growing season, they are usually confined to a small area, which is not efficient when dealing with a high-throughput analysis of biomass, and crop damage in the late growth stage could also be a concern in practical applications.

The rapid development of low-cost and relative easy to operate unmanned aerial vehicles (UAVs) provides a new means of remote sensing. They are more flexible than satellite-based remote sensing, and can overcome the survey area limitation of the ground-based platform. A UAV could fly at a low altitude and acquire an image at a high spatial resolution based on a pre-defined flight route. Different types of spectroscopic and image sensors for UAV have been developed, such as Red Green Blue (RGB) sensors, multispectral/hyperspectral imaging sensors, light detection and ranging (LiDAR) and infrared thermal imaging sensors, further extending UAV-based remote sensing to various applications. Previous studies have shown the potential of high resolution UAV-based RGB images for measuring plant height [2, 11, 12], biomass [13–15], vegetation fraction [16], plant density [17], and grain yield [18]. Due to the availability of the near-infrared (NIR) wavelengths in multispectral/hyperspectral images, spectral images have also become an alternative for UAV sensors in evaluating the physiological- and biochemical-related parameters of plants, such as leaf area index (LAI) [19, 20], vegetation fraction [16], flower fraction [21], nitrogen (N) status [22–24], net photosynthesis [25] and biomass [26]. Most of the reported studies applied a single sensor to estimate a specific trait of the crop. In recent years, with the requirement of collecting comprehensive information about plant growth status, more studies were focused on estimating plant growth-related traits by data fusion from different sensors [8, 27, 28]. Bendig et al. [14] utilized the canopy height extracted from the crop surface model (Hcsm) to estimate fresh aboveground biomass (FAGB) and dry aboveground biomass (DAGB) of barley with the coefficient of determination (r^2^) values of 0.72 and 0.68, respectively, and the result of the DAGB estimation was further improved with r^2^ of 0.80–0.82 by adding NIR vegetation indices (VIs) obtained from the ground-based spectral measurement. Wang et al. [29] proposed fusion of airborne LiDAR and hyperspectral data derived from two platforms to estimate DAGB of maize with the r^2^ and root mean square error (RMSE) of 0.88 and 0.32 kg/m^2^, respectively, and concluded that sensor fusion provided a better estimate of DAGB compared with the result obtained from LiDAR or hyperspectral data alone. More recently, Maimaitijiang et al. [30] used multi-sensor data collected from RGB, multispectral and thermal cameras that were mounted on different UAVs to estimate FAGB and DAGB of soybean, and reported that multispectral and thermal data fusion provided the best result for biomass estimation. The most studies as reviewed above mainly focused on estimating biomass based on the sensor data collected from different remote sensing platforms, which could add more uncertainty of the sensor data due to the variable illumination conditions and the systematic variability of the platforms during data acquisition. Furthermore, canopy coverages and structures of the crop vary at different growth stages, which would significantly affect the spectral characteristics and 3D point clouds extracted from multispectral and RGB images, respectively.

In this study, we developed a compact UAV with low-cost, lightweight dual image-frame snapshot cameras for dynamic monitoring of rice biomass at different growth stages. The specific goals were to: (1) investigate the spatial and temporal variations in UAV variables and aboveground biomass (AGB) under different N treatments and growth stages; (2) develop random forest (RF) model for AGB and panicle biomass (PB) estimations by using UAV aerial images and test variable importance for AGB and PB estimations; and (3) perform statistical analysis to evaluate the accuracy and robustness of the AGB and PB prediction models developed from the fusion of RGB and multispectral images.

## Methods

### Experimental site

The experimental site was located at the Grain-production Functional Area of Anhua Town, Zhuji City, Zhejiang Province, China (29°31′5.35″N, 120°6′6.12″E). It has an average altitude of 16 m above sea level, and the average annual temperature is 16.3 °C. Rice (Yongyou 1540) was cultivated in an experimental site of 25 subplots with 18 × 10 m^2^ of each, and they were treated with five levels of N fertilizers (0, 72, 120, 240 and 360 kg N/ha) with five repetitions. N fertilizers were applied in the form of urea with the rates of 40, 30 and 30% at the stages of preplanting, tillering, and booting, respectively. In addition, phosphate (P) fertilizer (120 kg/ha) and potash (K) (240 kg/ha) were applied in the form of Ca(H_2_PO_4_)_2_∙H_2_O and KCl, respectively, at the preplanting stage. Rice was transplanted in early June and harvested in middle to late October in 2017. A protected planting area, with a width of 1 m, was provided around the entire experimental site.

### UAV-based image data collection

**Fig. 1:**
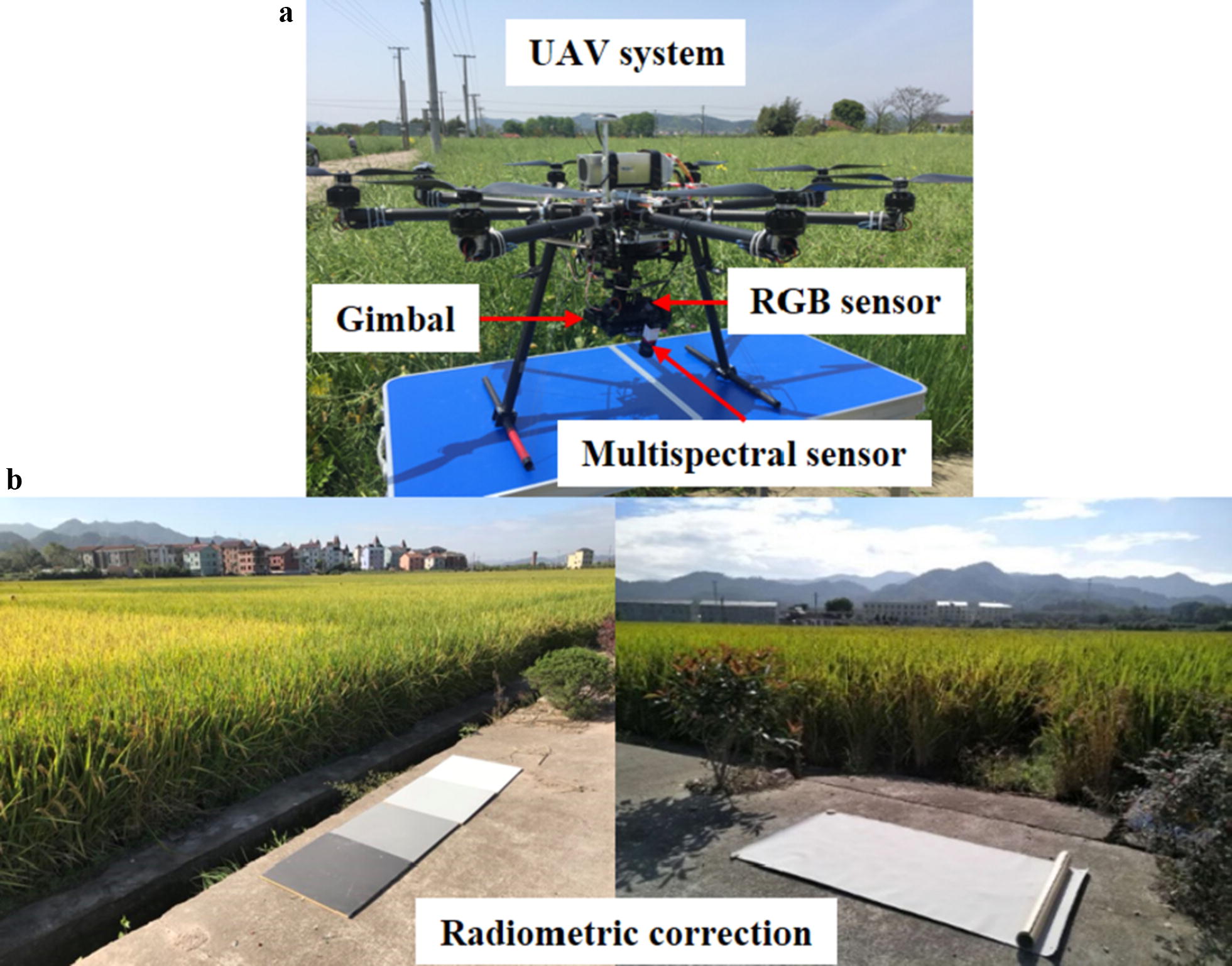
Illustration of the UAV system and radiometric calibration targets

An octorotor lightweight UAV, developed by the Digital Agriculture and Agricultural Internet-of-things Innovation Laboratory at Zhejiang University, was used to carry the image sensors (Fig. 1a). The UAV is 1.1 m in diameter and 0.35 m in height, and it has the maximum payload and the flight duration of 8 kg and 30 min, respectively. An RGB camera (NEX-7 camera, Sony, Dugang District, TKY, Japan) with a spatial resolution of 6000 × 4000 pixels and a snapshot multispectral camera (CMV2 K CMOS, IMEC, Chatsworth, Leuven, Belgium) with a spatial resolution of 409 × 216 pixels coupled with a three-axes gimbal were mounted on the UAV. The ground resolutions of RGB and multispectral cameras are 6 mm/pixel and 4.3 cm/pixel, respectively. The multispectral camera contains 25 wavelengths in the spectral region of 600–1000 nm (679, 693, 719, 732, 745, 758, 771, 784, 796, 808, 827, 839, 84, 860, 871, 880, 889, 898, 915, 922, 931, 937, 944, 951 and 956 nm). The UAV campaigns were conducted between 14:00 and 16:00 local time on 28 July (initial jointing stage), 28 August (initial heading stage), 18 September (initial filling stage) and 10 October (late filling stage), in 2017, and the weather was sunny without much wind. The GPS-controlled flight route was predefined with the autopilot mode in the flight control system. Fourteen ground control points (GCPs) were evenly distributed in the field as shown in Additional file 1: Figure 1S. The position of each GCP was measured using a GPS measuring instrument (iRTK2, HITARGET, Guangzhou, GD, China), which was used for image mosaicking, geometric correction and identification. The altitude for image acquisition was 25 m, with a flight speed of 2.5 m/s. The exposure times of RGB and multispectral cameras were adjusted based on the illumination conditions measured by a photometer (MQ-200, Apogee instruments, Logan, UT, USA). The flight route was planned with 60% and 75% lateral and forward overlaps, respectively, to achieve a good image mosaicking performance.

### Ground measurements of canopy height and aboveground biomass

After UAV campaigns, field measurements were conducted within 1 day. The ground truth data of the plant canopy height (Hcanopy), AGB and PB was collected from five 0.2 × 0.3 m^2^ zones in each plot. The sampling points were randomly selected, and the sampling positions were also recorded. The calculated height was the average of the determined height area. The rice canopy height between the ground and the highest point of the plant was measured in each subplot by using a ruler in the field at initial jointing and initial heading stages. Since the height of the plant canopy remained unchangeable when rice plants entered into the heading stage, no measurement of Hcanopy was performed after the heading stage. Then, five samples in the five quadrats were manually harvested from each subplot, and 500 sample points were obtained during the entire experiment to measure the ground truth of the biomass with four growth stages. These plants were sealed in plastic bags and taken to the laboratory within 6 h after harvesting. After transportation to the laboratory, plant samples were cleaned to remove the soil and water, and the roots of the plants were cut. Theses samples were then dried for 72 h, until a consistent weight was obtained. Finally, AGB with the weight per unit area (kg/m^2^) was calculated [14]. Meanwhile, PB was also measured at initial filling and late filling stages.

### Image processing

#### Crop surface models extraction

**Fig. 2:**
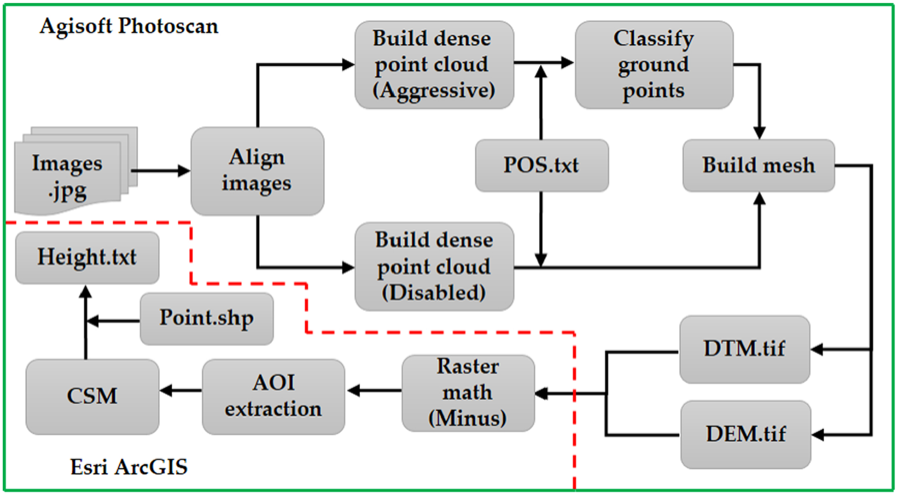
Workflow for crop surface model extraction and rice height estimation

Image mosaicking was first conducted using Agisoft PhotoScan Professional Software (Agisoft LLC, St. Petersburg, Russia), which uses matching features in the images to perform a bundle adjustment and generates a point cloud [31]. Based on the mosaicked RGB image, the crop surface model (CSM) was developed to determine the crop height [32, 33]. The point clouds were first generated using the structure from motion (SfM) method, and the detailed procedure can be found in the study of Tomasi et al. [34]. The point cloud consisted of the matched points between overlapping images, including crop canopy and terrain surfaces. By conducting the classification of point cloud, the digital elevation model (DEM) and the digital terrain model (DTM) were obtained. The DEM was generated based on the complete dense point clouds representing the height of the crop canopy, while the DTM was only developed from the dense point clouds of the ground surface. By importing two models into Esri ArcGIS software (ArcGIS, Esri.Inc, Redlands, CA, USA), the CSM can be obtained by subtracting the DTM from the DEM. For height information, a series of sampling points were defined around sampling area, and the elevation information for each point was then exported into a text file. Finally, the height data for each sampling point was determined, which was then fused with spectral VIs for the biomass estimation. The detailed workflow for CSM generation was shown in Fig. 2.

#### Radiometric and spectral correction

Radiometric calibration was first performed by five reference targets with a known reflectance measured by a ground-based spectrometer (QE65000, Ocean Optics, Dunedin, FL, USA) (Fig. 1b). The correction factors were calculated using the reflectance targets with the known reflectance and the digital number (DN) obtained from the onboard RGB and multispectral cameras, which were then used to transform the DN values of crop images into the reflectance based on the following equations:

1 $$R_{{\left( {i,k} \right)}} = DN_{{\left( {i, k} \right)}} \times a_{k} + b_{k} \left( {i = 1,2,3,4,5} \right)$$

2 $$\left( {\begin{array}{*{20}c} {R_{{\left( {1, k} \right)}} } \\ {R_{{\left( {2,k} \right)}} } \\ {R_{{\left( {3,k} \right)}} } \\ {R_{{\left( {4,k} \right)}} } \\ {R_{{\left( {5,k} \right)}} } \\ \end{array} } \right) = \left( {\begin{array}{*{20}c} {DN_{{\left( {1, k} \right)}} } \\ {DN_{{\left( {2,k} \right)}} } \\ {DN_{{\left( {3,k} \right)}} } \\ {DN_{{\left( {4,k} \right)}} } \\ {DN_{{\left( {5,k} \right)}} } \\ \end{array} } \right) \times a_{k} + b_{k}$$

where $R_{{\left( {i,k} \right)}}$ and $DN_{{\left( {i,k} \right)}}$ are the reflectance and DN values of the calibration target *i* in band *k*, respectively, and $a_{k}$ is the slope or gain and $b_{k}$ is the intercept or the offset [35]. An example of the result for estimating $a_{k}$ and $b_{k}$ at 796 nm was shown in Additional file 2: Figure 2S. Due to physical constraints of multispectral sensor, spectral correction was also performed to eliminate the negative effect on spectral information caused by the second order response, spectral leaking and crosstalk [26].

#### Vegetation indices calculation

Various VIs extracted from RGB images (RGB-VIs) and multispectral images (MS-VIs) have been used to evaluate the plant growth status. Nine VIs, as shown in Table 1, were calculated from RGB and multispectral images, which possess the capacity to estimate biomass. The calculated VIs were averages of the corresponding sampling areas in RGB and multispectral images, which were calculated based on the true sampling areas and ground resolutions. RGB-VIs are sensitive to the plant greenness, and they have been employed to extract green vegetation and calculate vegetation coverage such as visible-band difference vegetation index (VDVI), normalized green–red difference index (NGRDI), visible atmospherically resistant index (VARI), green–red ratio index (GRRI), and vegetativen (VEG) [16, 18, 36–38]. The modified VARI [MVARI = (G − B)/(G + R − B)] was also explored. Importantly, the RGB-VIs of modified green blue vegetation index (MGRVI), NGRDI, VDVI and VEG have been demonstrated many advantages on biomass assessment [2, 14].

**Table 1:** Vegetation indices (VIs) derived from red green blue (RGB) and multispectral images

Vegetation indices	Definition	References
*RGB*-*VIs*		
Visible-band difference vegetation index	VDVI = (2 * G − R − B)/(2 * G + R + B)	[37]
Normalized green–red difference index	NGRDI = (G − R)/(G + R)	[43]
Visible atmospherically resistant index	VARI = (G − R)/(G + R – B)	[38]
Green–red ratio index	GRRI = G/R	[44]
Vegetativen	VEG = G/(R^a^ * B^(1 − a)^) a = 0.667	[45]
Modified green blue vegetation index	MGRVI = (G^2^ − R^2^)/(G^2^ + R^2^)	[14]
*MS*-*VIs*		
Normalized difference spectral index	NDSI = (R_λ1_ − R_λ2_)/(R_λ1_ + R_λ2_)	[46]
Simple ratio index	SR = R_λ1_/R_λ2_	[47]
Modified normalized difference spectral index	MNDSI = (R_λ1_ − R_λ2_)/(R_λ1_ − R_λ3_)	[48]

R, G and B are the reflectance of Red, Blue and Green channels, respectively. R_λ1_ represents the reflectance of a variable band in the spectral region of 600–1000 nm. For an example, the NDSI_(796, 679)_ is calculated based on the reflectance data at λ_1_ = 796 nm and λ_2_ = 679 nm

**Fig. 3:**
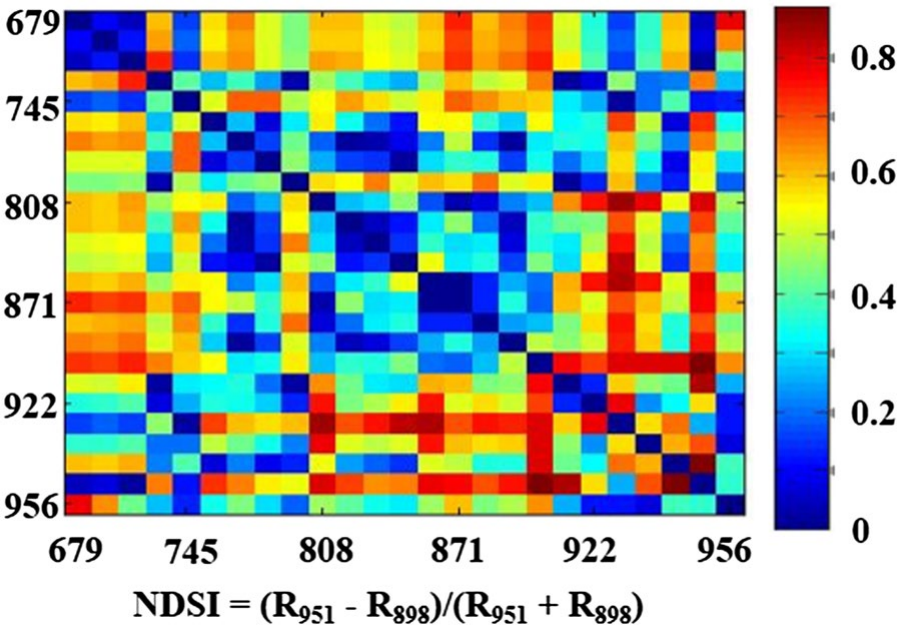
The contour map of the absolute r-value between aboveground biomass (AGB) and NDSI _(i, j)_, and it is calculated using reflectance values R_λ1_ and R_λ2_ at thorough combinations of two wavebands, i and j nm

MS-VIs can be classified into three categories: normalized difference spectral index (NDSI), simple ratio index (SR), and modified normalized difference spectral index (MNDSI). They were calculated using two or three available wavelengths in the spectral region of 600–1000 nm, which have been widely utilized to assess LAI, chlorophyll and N contents, biomass and grain yield [39–42]. Relative to the RGB-VIs that consist fixed wavelength combinations, the MS-VIs are determined with the optimal wavelength combinations at the given spectral region of multispectral images. In this study, the r^2^ between biomass (AGB and PB) and MS-VIs (SR, NDSI and MNDSI) were first calculated using all combinations of any wavelengths to select the significant index with optimized wavelengths. In this article, the r^2^ values of all possible wavelength combinations were presented using the contour map as shown in Fig. 3. The combinations presenting the highest r^2^ to rice biomass were selected, which had a higher predictive ability.

### Statistical analysis and model development

The spatial heterogeneity and temporal variation in the typical VIs including the NDSI_(796, 679)_ and VDVI, and AGB were first investigated. The NDSI_(796, 679)_ was equivalent to the normalized difference vegetation index (NDVI), which was closely related to the canopy greenness, N content, aboveground N uptake, and N efficiency of crops [49]. VDVI was also a good indicator of crop growth, canopy greenness and yield [18]. Therefore, NDSI_(796, 679)_ and VDVI values can well reflect seasonal changes in phenology of rice. Based on RGB images and multispectral reflectance images, the VDVI and NDSI_(796, 679)_ maps were produced using the equations as shown in Table 1. The average values of VDVI and NDSI_(796, 679)_ for each plot were calculated to represent the average growth condition. Then, the inter-correlations among all of the UAV variables, including the Hcsm, RGB-VIs, and MS-VIs, were evaluated using Pearson correlation coefficient (r). Furthermore, a regression analysis was performed to investigate the feasibility of VIs and Hcsm to estimate AGB and PB.

Considering the possible nonlinear relationships between these UAV variables and biomass, the RF model that can identify the collinear and nonlinear relationships among variables was proposed. The RF model can handle a large number of variables and assess the importance of each variable. It was reported that the generalization performance and the training efficiency of the RF model were both improved compared with the stepwise regression (SWR) and the back propagation neural network (BPNN) methods [50, 51]. RF model utilized the bagging method, which creates a separate tree using a random sample of the data set to estimate variable importance with the following equation:

3 $$Importance \left( X \right) = \mathop \sum \limits_{i = 1}^{n} \frac{errOOB2 - errOOB1}{n}$$

where *errOOB1* represents the error of out of bag for variable *X* with one decision tree, *errOOB2* represents the error of adding noise to variable *X* with one decision tree, and *n* represents the number of decision trees.

During the model development, the dataset was divided into a training set (2/3) and a testing set (1/3), with a ten-fold cross-validation to reduce the variability of the modeling. The model performance was evaluated using the r^2^, relative root mean square error (RRMSE) and the root mean square error of prediction (RMSEP) [2]. A higher r^2^ and a lower RMSEP and RRMSE indicate a better estimation performance. The mean absolute deviation (MAE) was also used to evaluate the distribution of error around the mean of data. Meanwhile, to investigate the response of the change of model performance to perturbations in the input parameters and simultaneously provide a theoretical basis for simplifying the model, sensitivity analysis was also performed [52]. It determines the model result changes when the model parameters are changed. The parameters were removed one by one to re-simulate the prediction of the model while keeping the other parameters unchanged. Finally, the sensitivity, MAE, RMSEP and RRMSE were calculated as follows:

4 $$Sensitivity = \frac{{r_{i}^{2} - r^{2} }}{{R^{2} }} \times 100\%$$

5 $$MAE = \frac{1}{n}\mathop \sum \limits_{1}^{n} |p_{i} - \hat{p}_{i} |$$

6 $$RMSEP = \sqrt {\frac{1}{n}\mathop \sum \limits_{1}^{n} \left( {p_{i} - \hat{p}_{i} } \right)^{2} }$$

7 $$RRMSE = \frac{RMSE}{{\bar{p}_{i} }} \times 100\%$$

where *r*^*2*^ and *r*_*i*_^*2*^ represent the coefficient of determination based on a tenfold cross-validation of the original prediction model and the re-simulated prediction model, respectively, by removing the parameter *i*, which is the number of input parameters. In addition, $p_{i}$ is the measured value, $\bar{p}_{i}$ is mean value of all measured values and $\hat{p}_{i}$ is the predicted value.

## Results

### Spatial–temporal variations in NDSI_(796, 679)_, VDVI and AGB

**Fig. 4:**
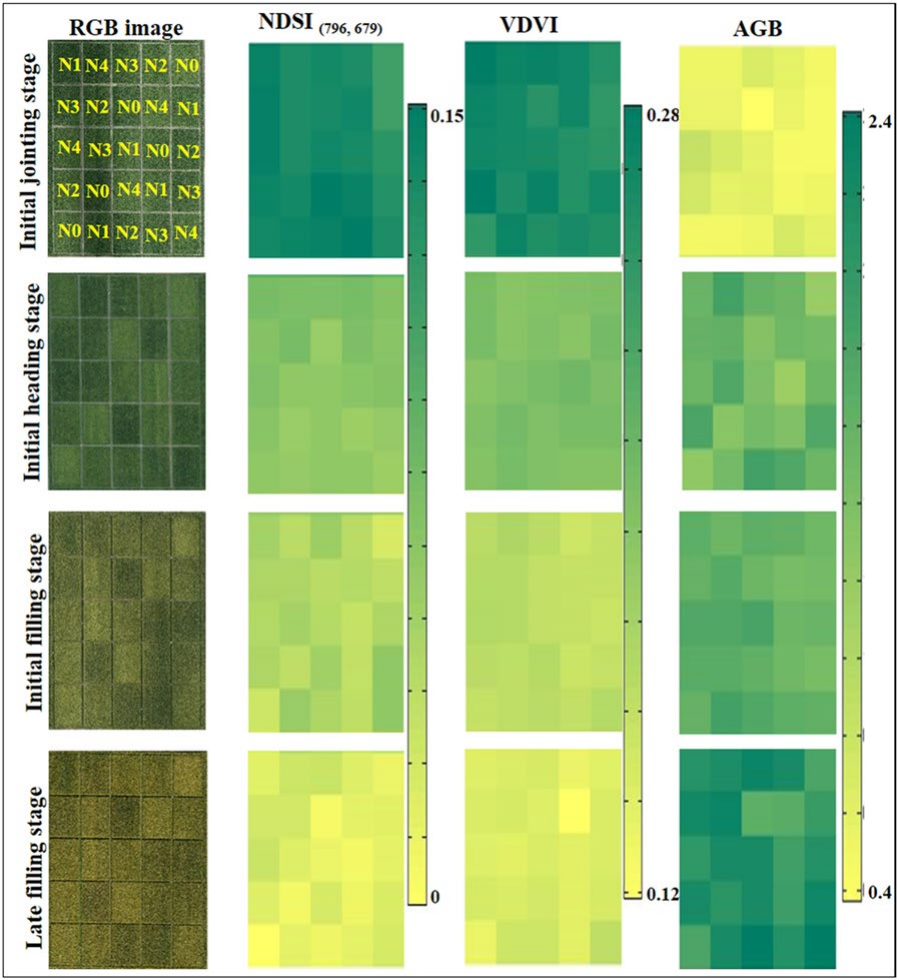
Spatial and temporal variations in Red Green Blue (RGB) images, NDSI_(796, 679)_, VDVI and AGB (kg/m^2^) of rice. NDSI_(796, 679)_, VDVI, AGB and N represent the normalized difference spectral index, visible-band difference vegetation index, aboveground biomass, and nitrogen fertilizers

The spatial and temporal variations in RGB images, NDSI_(796, 679)_ and VDVI as well as AGB of the rice during growing stages in the experimental field, are shown in Fig. 4. The growth differences among plots with different N treatments were visually observed from the RGB images, providing an intuitive view on the change of canopy greenness with a tendency from green to yellow. It was found that the increased N application rates had a positive effect on the NDSI_(796, 679)_, VDVI and AGB at the four growth stages. From the initial jointing stage to the late filling stage, NDSI_(796, 679)_ and VDVI showed a significant decreasing tendency in similar with the change of canopy greenness, while AGB maintained the growth trend.

### Canopy height derived from crop surface model

**Fig. 5:**
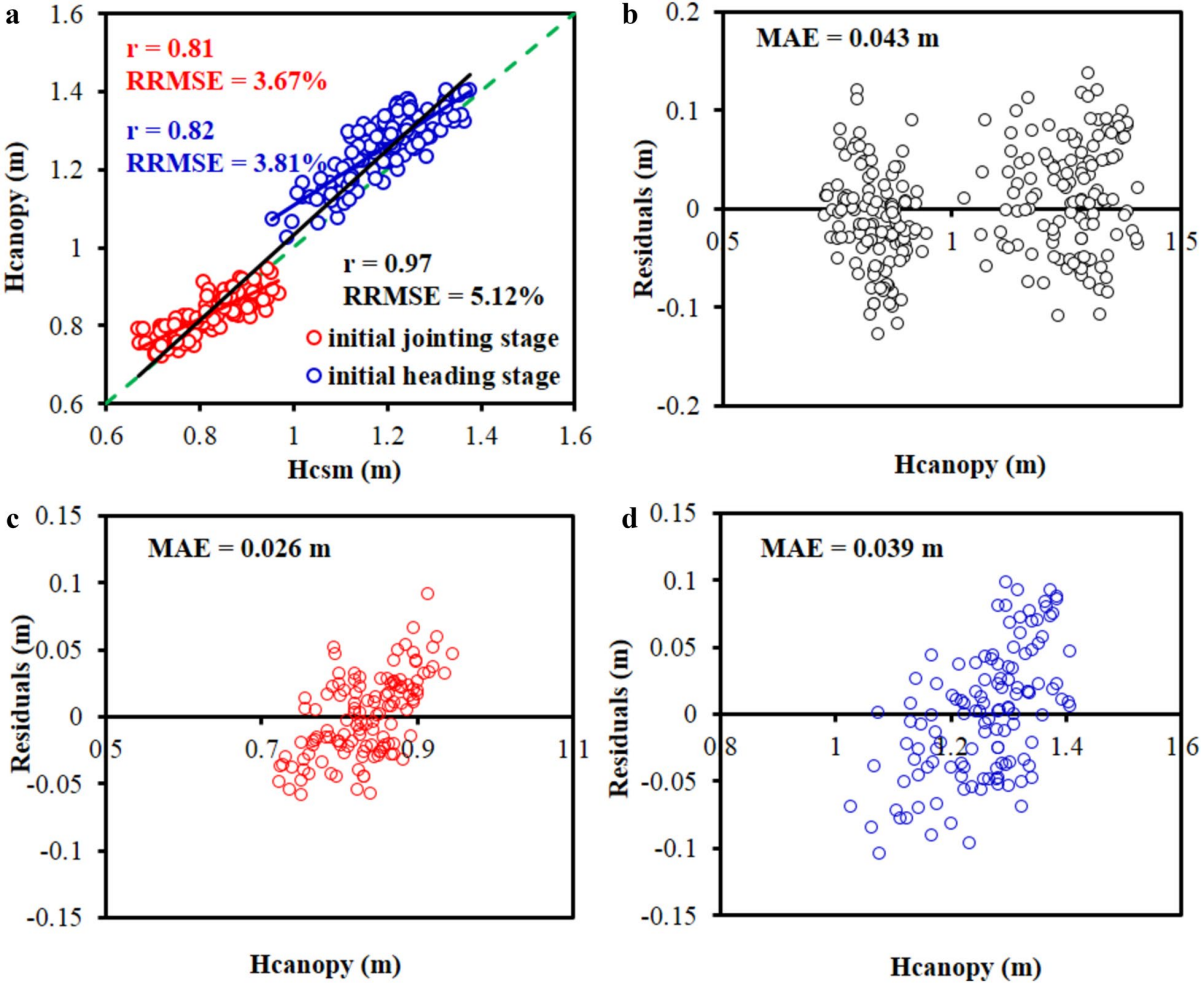
Correlations between the canopy height derived from crop surface model (Hcsm) and canopy height (Hcanopy) from field measurements at the initial jointing and initial heading stages (**a**), and residual plots of the error distributions for **b** two stages, **c** initial jointing stage, and **d** initial heading stage. The r, RRMSE and MAE represent the Pearson correlation coefficient, relative root mean square error, and mean absolute deviation, respectively

**Fig. 6:**
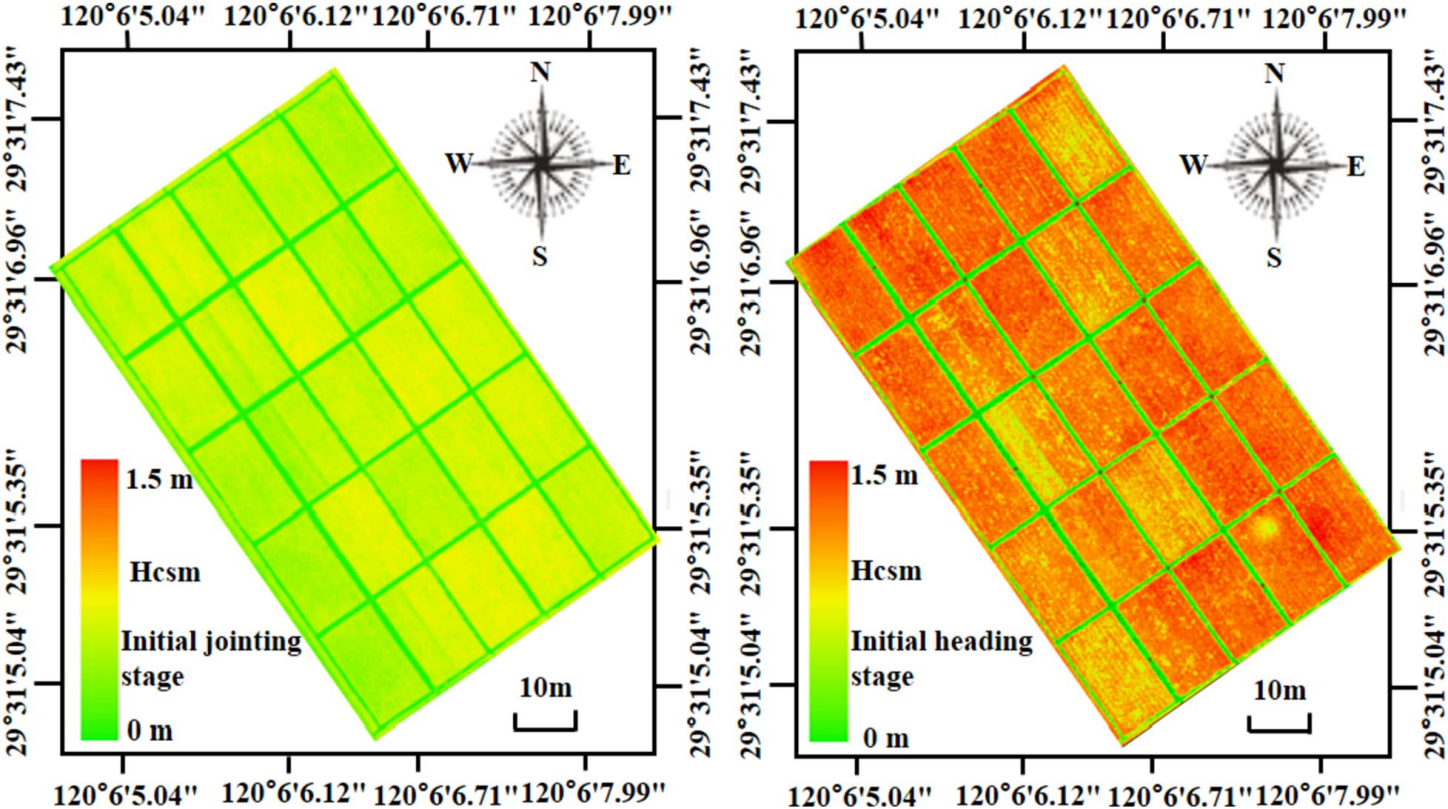
Distributions of canopy height derived from crop surface model (Hcsm) developed from Red Green Blue (RGB) images acquired at the initial jointing and initial heading stages. Coordinates are displayed in the World Geodetic System 1984 Coordinate System

Figure 5a presents the correlations between Hcsm and Hcanopy at the initial jointing and the initial heading stages, and a high correlation was observed with the r, RRMSE, and MAE of 0.97, 5.12% and 0.043 m, respectively. Considering the correlations at individual stages, the r significantly decreased with the values of 0.81 and 0.82 for the initial jointing and initial heading stages, respectively, due to the relative narrow distribution of the height data, but the MAE also decreased (Fig. 5b–d). The lowest RRMSE of 3.67% was obtained between the Hcsm and Hcanopy at the initial jointing stage. As shown in Fig. 6, the distribution maps of CSM produced from UAV-based RGB images clearly presented that there existed differences in Hcanopy among different plots with an increasing tendency from low to high N treatments. These results not only presented the performance of CSM for a quantitative estimation of Hcanopy, but also provided a visualization of Hcanopy distributions on the entire field scale.

### Estimation of biomass during rice growth stages

#### Correlations for UAV variables and AGB

**Fig. 7:**
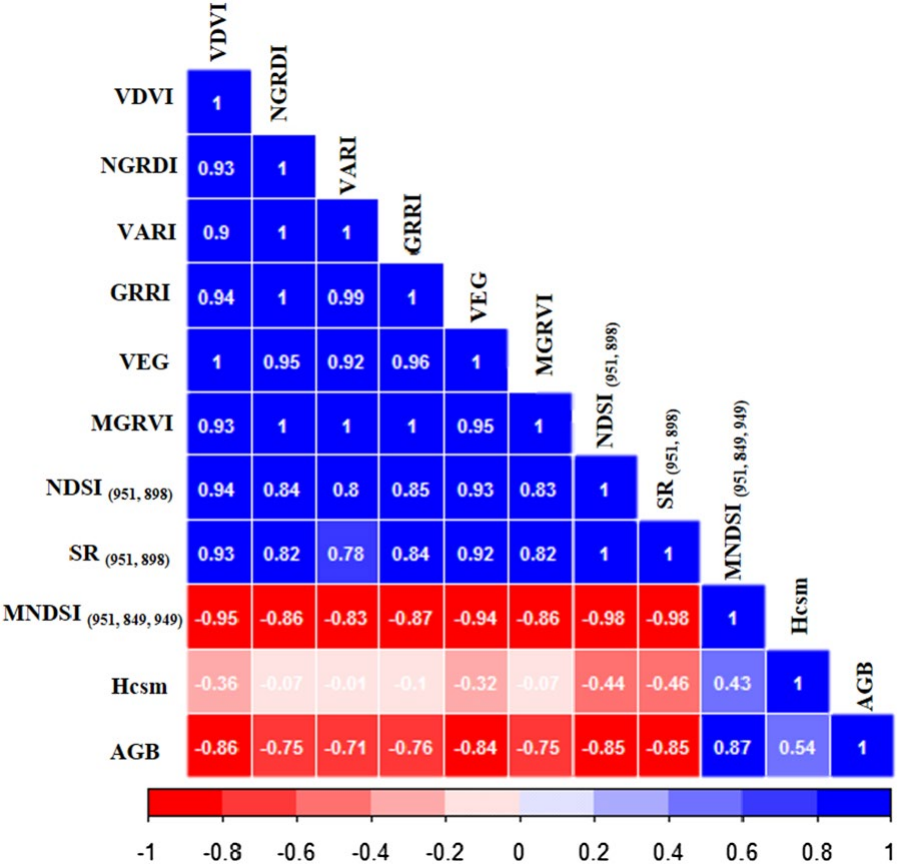
Correlation analysis (r) between aboveground biomass (AGB) and individual UAV variables from Red Green Blue (RGB) and multispectral images

Figure 7 shows the Pearson’s correlation among Hcsm, RGB-VIs, selected MS-VIs, and field-measured AGB at four growth stages. The highest correlation was found between MNDSI_(951, 849, 949)_ and AGB, with the absolute r-value of 0.87 followed by VDVI (r = 0.86). This also confirmed that MNDSI_(951, 849, 949)_, SR_(951, 889)_, NDSI_(941, 889)_ and VDVI were promising indicators for AGB estimation in this field experiment. While the Hcsm had a relative low correlation with biomass (r = 0.54), indicating the limitation of Hcsm for AGB estimation during the entire growth stages. Additionally, high correlations also existed among several VIs such as RGB-VIs of NGRDI, VARI, GRRI and MGRVI and three MS-VIs.

#### Development of AGB estimation model

**Fig. 8:**
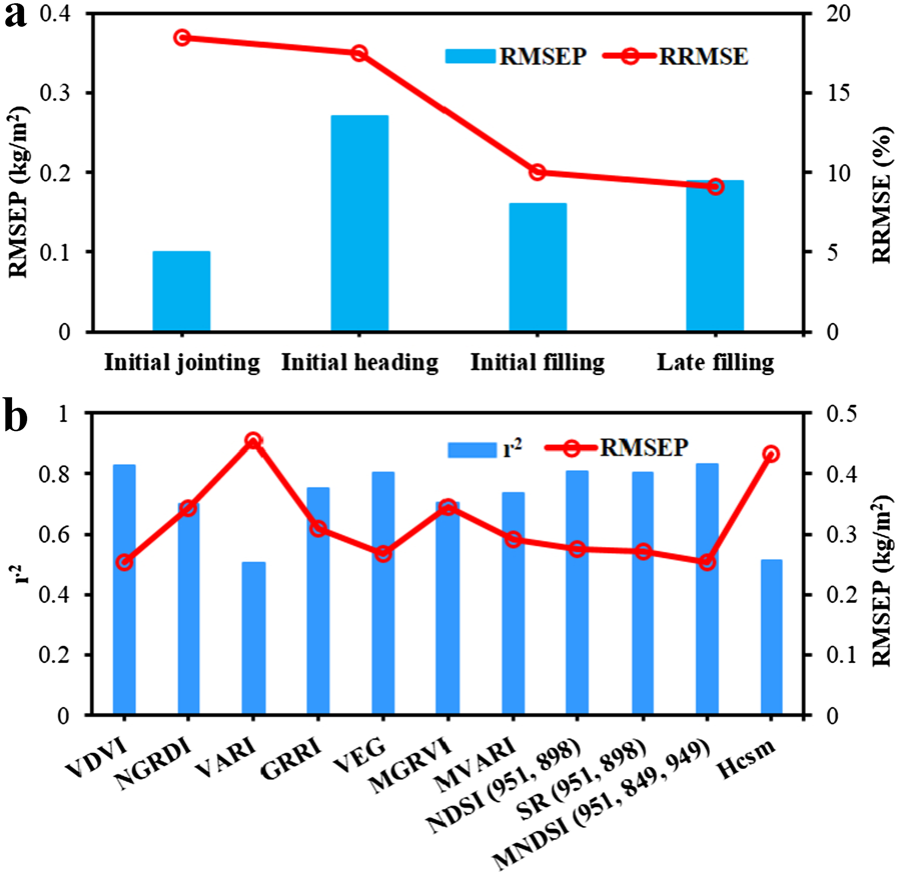
Estimations of aboveground biomass (AGB) at different growth stages by random forest (RF) model using the combination of nine vegetation indices (VIs) and canopy height derived from crop surface model (Hcsm) (**a**). Estimation of biomass developed by RF model using single variable with four growth stages (**b**). The r^2^, RMSEP and RRMSE represent the coefficient of determination, the prediction of root mean square error and relative RMSE, respectively

**Fig. 9:**
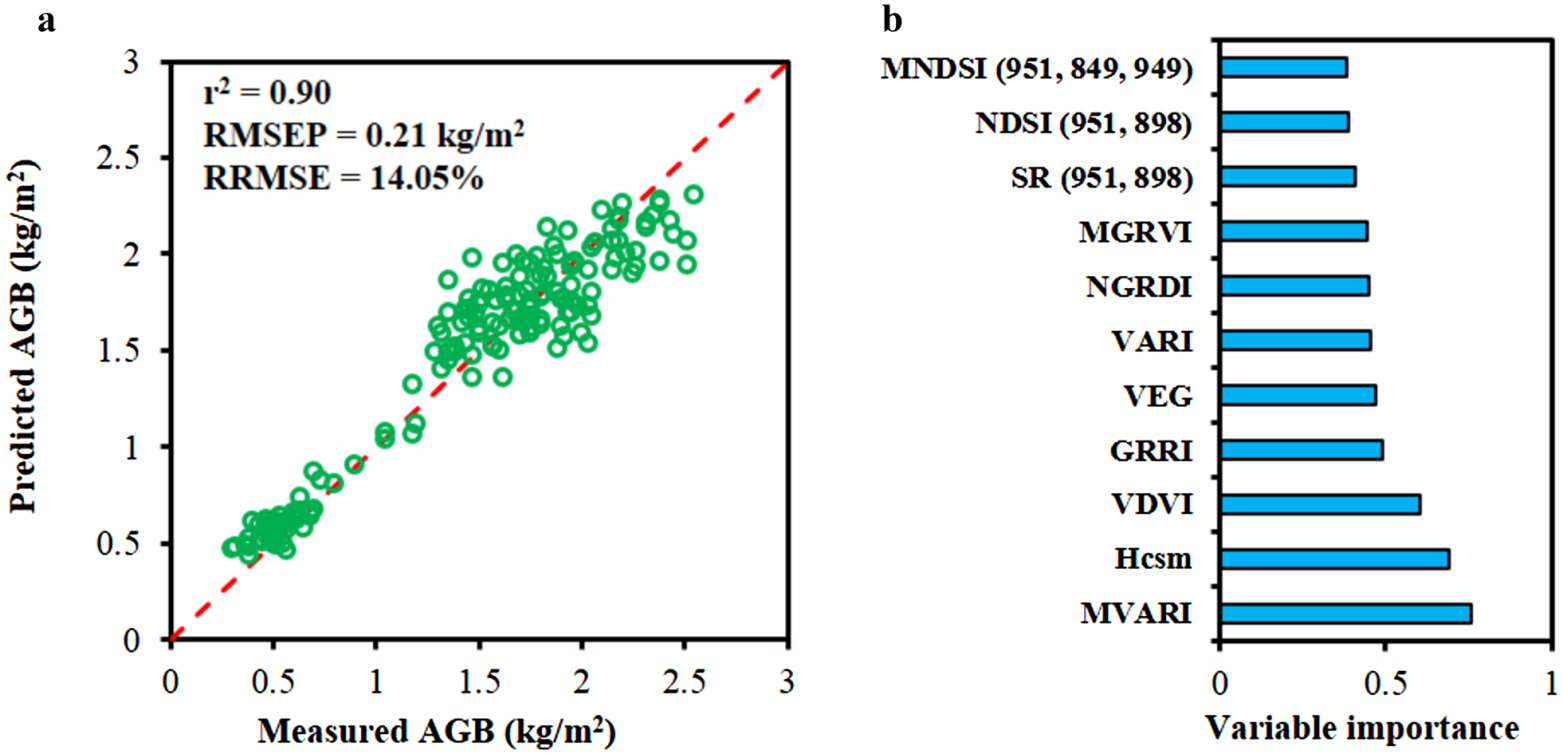
Estimation of aboveground biomass (AGB) using random forest (RF) model developed from UAV variables extracted from Red Green Blue (RGB) and multispectral images (**a**). Dashed red line is the 1:1 line. The right figure shows the variable importance estimation of the RF model (**b**). The r^2^, RMSEP and RRMSE represent the coefficient of determination, the prediction of root mean square error and relative RMSE, respectively

Based on the correlation analysis, RF model for AGB prediction was developed using the combinations of Hcsm, RGB-VIs and MS-VIs extracted from RGB and multispectral images. Figure 8a shows the RMSEP and RRMSE values for biomass estimations at different growth stages, and the smallest RMSEP and RRMSE were obtained at initial jointing and late filling stages, respectively. The RRMSE values of these variables consistently increased from the initial jointing stage to the late filling stage, indicating that AGB estimation possessed relatively smaller errors when rice gradually became mature. For nine VIs and Hcsm versus biomass with four growth stages (Fig. 8b), the best prediction for AGB was achieved by MNDSI_(951, 849, 949)_ (r^2^ = 0.83 and RMSEP = 0.25 kg/m^2^). In addition, VDVI, VEG, SR_(951, 898),_ and NDSI_(951, 898)_ also exhibited a high r^2^ of above 0.8, while VARI and Hcsm showed relative lower r^2^ values of 0.50 and 0.51, respectively. Further combination of nine VIs and Hcsm at four growth stages achieved the best AGB prediction with the r^2^, RMSEP and RRMSE of 0.90, 0.21 kg/m^2^ and 14.05%, respectively (Fig. 9a), which indicated that fusion of dual-camera image data improved the estimation of AGB. Based on the analysis of the variable importance in RF model shown in Fig. 9b, it was found that RGB-VIs were more valuable for AGB prediction than MS-VIs in general, and the Hcsm showed the highest variable importance.

#### Estimation of PB at the mature phase

**Fig. 10:**
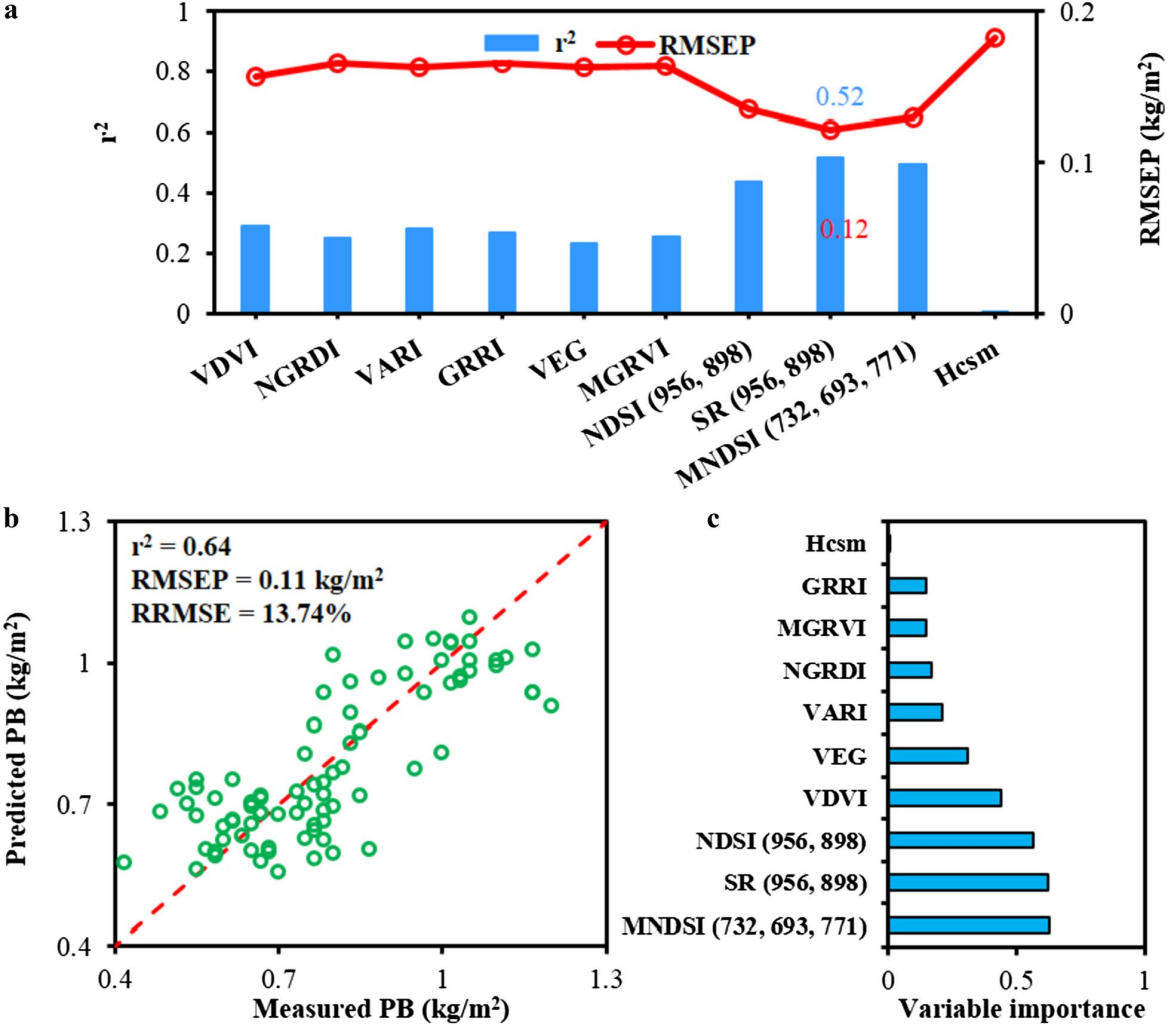
The r^2^ for nine vegetation indices (VIs) and canopy height derived from crop surface model (Hcsm) versus panicle biomass (PB) (**a**). Estimation of panicle biomass using random forest model developed from UAV variables extracted from Red Green Blue (RGB) and multispectral images (**b**). Dashed red line is the 1:1 line. The right figure shows the variable importance estimation of the RF model (**c**). The r^2^, RMSEP and RRMSE represent the coefficient of determination, the prediction of root mean square error and relative RMSE, respectively

At the mature phase (initial filling and late filling stages), the estimation of PB was also conducted, which was closely related to the final rice yield. To show above variables’ ability to estimate PB of rice at the mature phase, the estimation results developed by individual UAV variables were shown in Fig. 10a. Among all variables, SR_(956, 898)_, NDSI_(956, 898)_ and MNDSI_(732, 693, 771)_ exhibited relatively good performance, which was consistent with the modeling of AGB as presented in Fig. 8b. This suggested that MS-VIs possessed relative higher capacity of estimating AGB and PB. The PB prediction result of RF model with all UAV variables was shown in Fig. 10b, and a reasonable accuracy was obtained with r^2^, RMSEP and RRMSE of 0.64, 0.11 kg/m^2^ and 13.74%, respectively. This suggested that dual-camera data fusion could improve the result of PB estimation. As expected, SR_(956, 898)_, NDSI_(856, 898)_ and MNDSI_(732, 693, 771)_ presented relatively high variable importance to estimate PB, and it also pointed that MS-VIs possessed more value for PB prediction than RGB-VIs (Fig. 10c).

#### Comparison of RGB and multispectral cameras for biomass estimation

To assess the predictive capabilities of different cameras, the r^2^, RMSEP and RRMSE were calculated for evaluating the model performances. Based on RF estimates of AGB and PB, the multispectral and RGB image data provided the comparable result for AGB estimation, and the multispectral image data, which included the NIR spectral region, outperformed the RGB image data for PB estimation (Table 2). Further examination of sensor fusion showed that the combination of RGB and multispectral image data presented the best estimations for AGB and PB with the smallest RRMSE of 14.05% and 13.74%, respectively. Compared to the results obtained from the individual sensors, the values of RMSEP of AGB and PB were reduced by 8.33–16.00%, which indicated that fusion of RGB and multispectral image data can substantially improve the biomass estimations.

**Table 2:** Estimated aboveground biomass (AGB) and panicle biomass (PB) in rice by random forest (RF) method

Camera type	Features	Estimation	AGB	PB
RGB	VDVI, NGRDI, VARI, GRRI, VEG, MGRVI, Hcsm	r^2^	0.85	0.48
		RMSEP	0.23	0.13
		RRMSE	15.57%	16.93%
Multispectral	NDSI, SR, MNDSI	r^2^	0.83	0.53
		RMSEP	0.25	0.12
		RRMSE	17.02%	14.91%
RGB + multispectral	VDVI, NGRDI, VARI, GRRI, VEG, MGRVI, NDSI, SR, MNDSI, Hcsm	r^2^	0.90	0.64
		RMSEP	0.21	0.11
		RRMSE	14.05%	13.74%

The r^2^, RMSEP and RRMSE represent the coefficient of determination, the prediction of root mean square error and relative RMSE, respectively

## Discussion

In this study, we discussed a lightweight UAV equipped with dual image-frame snapshot cameras and the performance of estimating rice biomass (AGB and PB) by RGB and multispectral images under a field environment. The results have demonstrated the potential of fusing RGB and multispectral image data for biomass estimations.

Both RGB and multispectral cameras could provide spectral information in the visible spectral region, which was closely related to the vegetation greenness [16, 36]. While considering biomass estimation, RGB camera and multispectral camera possessed own advantages and disadvantages as presented in Figs. 7, 8, 9 and 10. Figure 9 revealed that the Hcsm and RGB-VIs of MVARI and VDVI possessed the higher importance for the assessment of AGB than MS-VIs. This may be due to that RGB images with a higher spatial resolution contained canopy structural information, resulting in obtaining relatively clear phenotypes of crops such as vegetation coverage and plant height, and surpassed the performance of the multispectral sensor in the spatial domain [2, 14, 16]. Moreover, RGB images can provide rich texture information, and the SfM technique with an RGB camera is able to generate denser point cloud data, and is thus suitable for restoring the intricate surface texture of plant structure [53]. Compared to RGB sensor, the multispectral sensor with a wider wavelength range could provide the NIR spectral information that reflects physiological characteristics of crops [30, 54], especially for estimating the panicle biomass as shown in Fig. 10a. However, the saturation issue associated with using the multispectral sensor in a dense vegetation canopy could be a limitation for the biomass estimation [30]. Hence, each sensor or data set could be both limited in accuracy and incomplete. Combining data from RGB and multispectral cameras provided a holistic view of the plant growth status, and it was also possible to increase the signal to noise ratio for the final estimation. Our results indicated that fusing RGB with multispectral image data did improve the prediction results of biomass as shown in Table 2, since both crop canopy structural features and diverse spectral characteristics with NIR wavelengths related to the crop biomass were introduced.

Agronomically, there are two growth phases of rice: vegetative and reproductive [55]. The vegetative phase refers to the period from germination to the initiation of panicle with four stages, including emergence, seedling development, tillering and internode elongation [55]. The first two growth stages describe the process from the emergence of the radicle to the onset of tillering, which were generally not considered in the field experiment due to the limited information of crop growth that current sensors can obtain. At tillering stage, rice plants were too small to present significant growth difference among different N applications, which was unsuitable for the prediction of biomass. In addition, matched points of the images extracted from the top canopy mixed with the lower parts of crops or soil background due to the sparse structure could also affect the plant height extraction from CSMs [11, 56]. When the tillering stage ends, the rice plant entered into the jointing stage, which has basically formed a continuous canopy that could contribute to extract height information from the CSM precisely. When rice plants entered into the initial heading stage, most of the plant nutrients were used to develop panicles, and there would be a less change in the plant height while the biomass was still accumulated. This indicated that the relationship between height and biomass varied with different growth stages, and therefore, it would be difficult to determine the biomass by only using the Hcsm when rice plants entered into mature stages, which was similar to the results shown in Figs. 8b and 10a.

Data analysis is another challenge in remote sensing since images obtained from remote sensing includes different noises and information is highly correlated. Effective machine learning methods are usually required to interpret the data and to develop robust prediction models. Taking account of the influence of different models which was also reported in the previous study [2], the performance of RF model was also compared with three regression models, including extreme learning machine (ELM), BPNN and least square-support vector machine (LS-SVM) (as shown in Table 3). In general, all the models generated reasonable results, and RF showed the best performance of the estimations of AGB and PB as well as LS-SVM for PB estimation.

**Table 3:** Estimations of aboveground biomass (AGB) and panicle biomass (PB)

Regression methods	AGB			PB		
	r^2^	RMSEP (kg/m^2^)	RRMSE (%)	r^2^	RMSEP (kg/m^2^)	RRMSE (%)
RF	0.90	0.21	13.56	0.64	0.11	14.14
ELM	0.87	0.22	15.42	0.64	0.11	14.16
BPNN	0.87	0.23	15.73	0.59	0.13	15.85
LS-SVM	0.89	0.21	14.64	0.64	0.11	14.14

RF, ELM, BPNN and LS-SVM represent random forest, extreme learning machine, back propagation neural network and least square-support vector machine. The r^2^, RMSEP and RRMSE represent the coefficient of determination, the prediction of root mean square error and relative RMSE, respectively

**Fig. 11:**
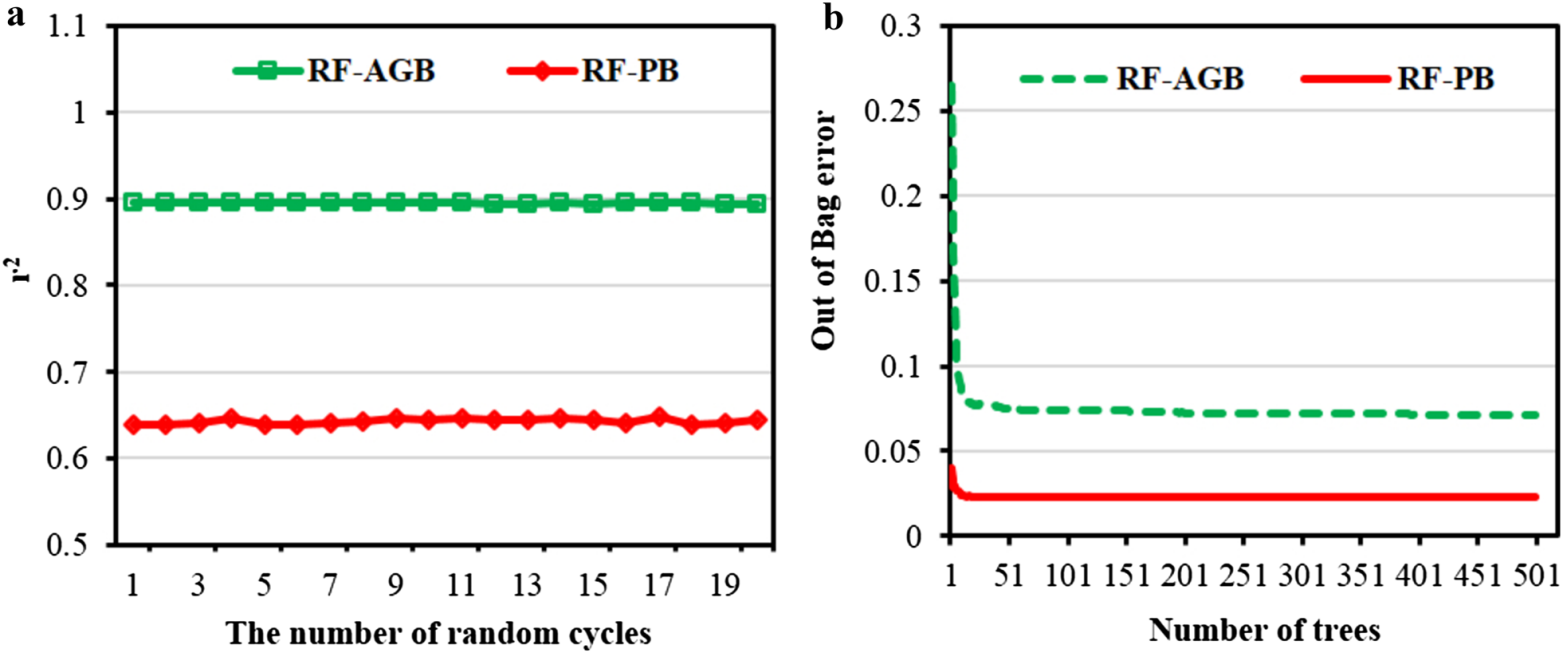
Stabilities of the random forest (RF) model for the estimations of aboveground biomass (AGB) and panicle biomass (PB) with the ten-fold cross-validation by using the r^2^ (**a**) and the out of Bag error (**b**)

The random selection of the training and testing sets was also one of the keys to improve the model performance. It is thus necessary to determine the stability of the prediction model by randomly dividing the dataset. As shown in Fig. 11a, it was observed that the RF model presented a stable performance when estimating AGB and PB. Consequently, we could conclude that the selected models were relatively reliable to conduct the prediction of rice biomass. In addition, the number of trees of RF model was crucial for the accuracy and the time cost of modeling. As shown in Fig. 11b, the out of Bag error reached the minimum and remained stable with the number of trees above 50 for AGB and PB estimation, and the prediction for PB developed by RF model possessed a smaller out of Bag error.

**Fig. 12:**
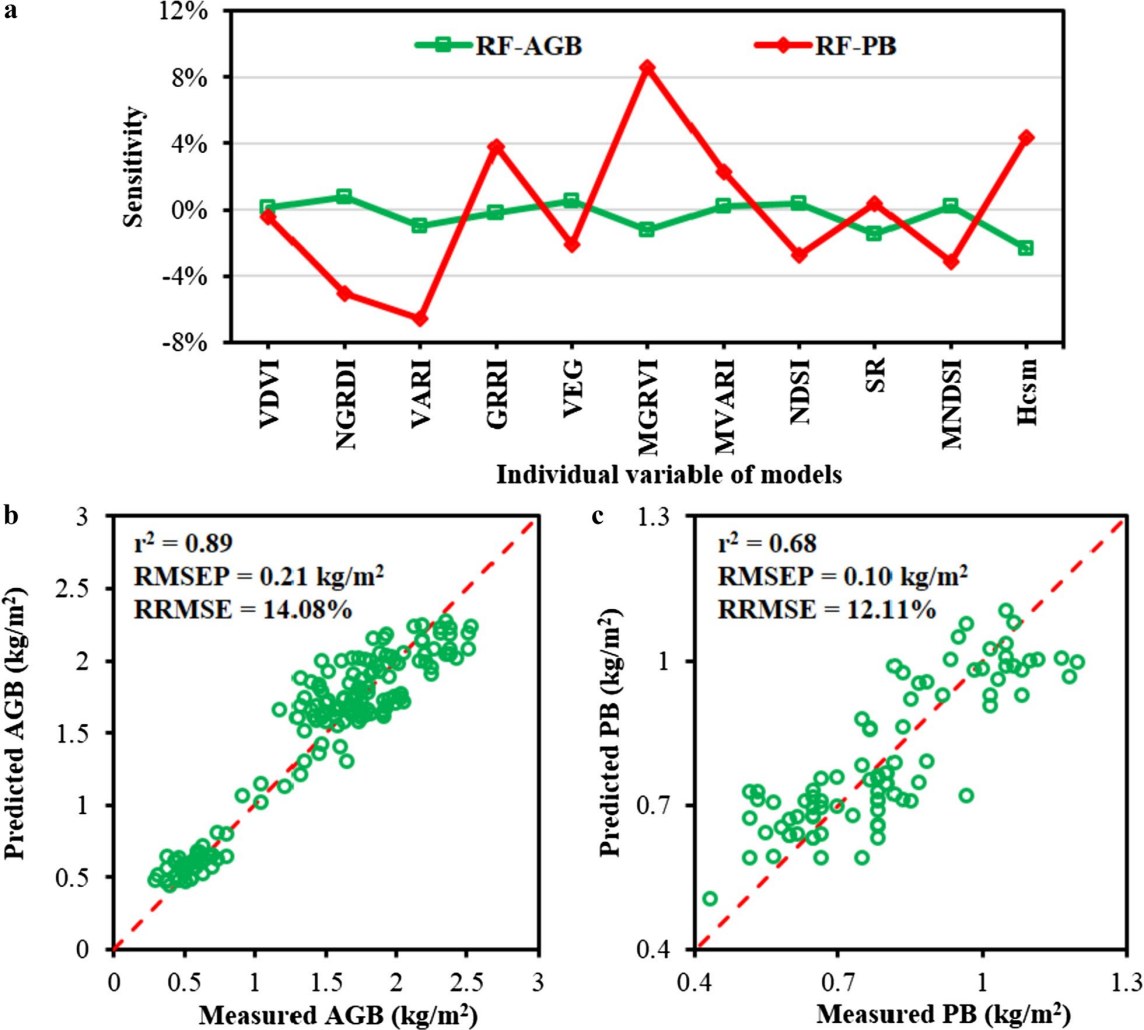
The sensitivity analysis of prediction models for aboveground biomass (AGB) and panicle biomass (PB) (**a**), and the prediction models for AGB (**b**) and PB (**c**) based on the sensitivity analysis using the variables with the sensitivity below zero. The r^2^, RMSEP and RRMSE represent the coefficient of determination, the prediction of root mean square error and relative RMSE, respectively

From mentioned above, the prediction model consisted of various UAV-based variables, while there existed large difference of correlations for VIs and Hcsm versus biomass. Consequently, it was critical to discuss the sensitivity of these variables to the entire prediction model, and the analysis result was shown in Fig. 12. The input variable with a sensitivity below zero indicated that the variable had a positive role in improving the prediction model. There did not exist high sensitivity of variables that could improve the estimation of AGB, while the sensitivity of variables to the PB estimation exhibited large difference. It was obvious that the MGRVI possessed the highest negative sensitivity to the prediction model compared with other variables, followed by the VARI. Further, new prediction models were developed based on the sensitive variables as shown in Fig. 12b, c. It was observed that fusion of RGB-VIs and MS-VIs improved the prediction results of PB with the combination of six sensitive variables including VDVI, NGRDI, VARI, VEG, NDSI_(856, 898)_ and MNDSI_(732, 693, 771)_ with the r^2^ and RMSEP of 0.68 and 0.10 kg/m^2^, respectively. Moreover, the estimation of AGB was implemented with the smaller variable combination of VARI, GRRI, MGRVI, SR_(951, 949),_ and Hcsm (Fig. 12c), and the prediction performance was comparable to the result that was obtained from RF model with all UAV variables as shown in Fig. 9a. It was concluded that variable sensitivity analysis could simplify the prediction model with achieving decent prediction results.

## Conclusions

This research demonstrated that a lightweight UAV with dual image-frame snapshot cameras has the potential for estimating biomass of rice during the entire growth stages. The spatial and temporal variations were observed in typical VIs (e.g., VDVI and NDSI_(796, 679)_), as well as AGB under different N treatments and growth stages. The correlation analysis between Hcsm and Hcanopy was conducted to verify the accuracy of the CSMs. We also examined the capacity of various UAV variables derived from UAV-based RGB and multispectral images to estimate AGB and PB. It was found that the Hcsm extracted from RGB images exhibited a high correlation with the ground-measured Hcanopy, while it was unsuitable to be independently used for biomass assessment of rice during the entire growth stages. MS-VIs showed higher correlations with AGB and PB than RGB-VIs. Compared with individual UAV variables, the performance of RF models developed by the fusion of RGB and multispectral image data was substantially improved for estimating AGB and PB. Moreover, RF models can be further simplified by sensitivity analysis while without reducing the prediction accuracy.

For the future work, it would be useful to improve the temporal resolution for the image acquisition of the crop in order to develop a continue plant growth model. Sophisticated data fusion algorithms and advanced machine learning methods would be helpful to improve the robustness and accuracy of prediction models for crop growth-related trait estimations. The UAV-based dual-sensor remote sensing platform will be further used to collect more rice growth-related traits in different cultivars and regions to develop a remote sensing database for rice.

## Additional files

**Additional file 1.** Location of the study area and detail of the experimental plot layout for the rice nitrogen treatments with ground control points (GCPs).

**Additional file 2.** Radiation calibration result at 796 nm.
