# Supplementary material for: Dynamic monitoring of biomass of rice under different nitrogen treatments using a lightweight UAV with dual image-frame snapshot cameras
Source: Plant Methods. 2019 Mar 27;15:32. doi: 10.1186/s13007-019-0418-8 (PMC6436235; doi:10.1186/s13007-019-0418-8)
Supplement: Supplementary file 1 — Additional file 1. Location of the study area and detail of the experimental plot layout for the rice nitrogen treatments with ground control points (GCPs). [file 13007_2019_418_MOESM1_ESM.pdf]

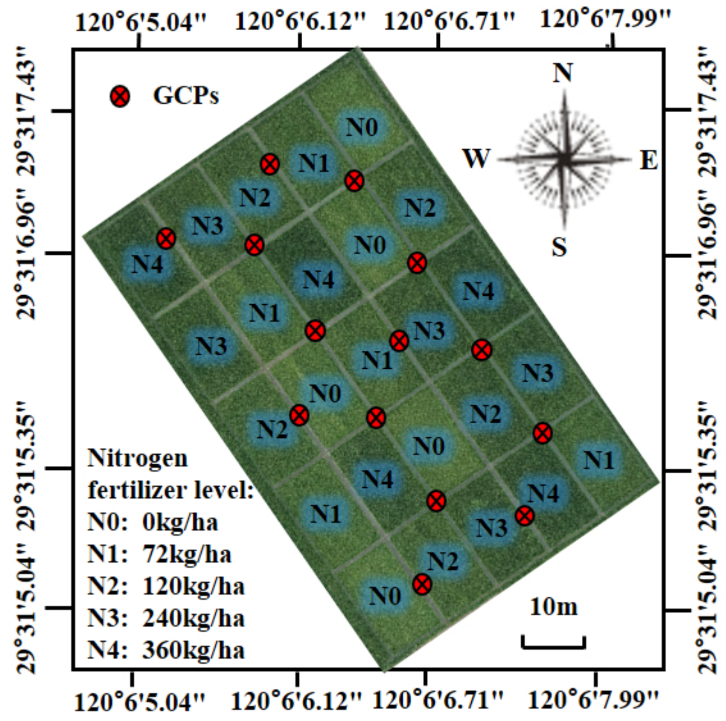

Fig. S1 Location of the study area and detail of the experimental plot layout for the rice nitrogen treatments with ground control points (GCPs).
