# Supplementary figures and images for: Dynamic monitoring of biomass of rice under different nitrogen treatments using a lightweight UAV with dual image-frame snapshot cameras
Source: Plant Methods. 2019 Mar 27;15:32. doi: 10.1186/s13007-019-0418-8 (PMC6436235; doi:10.1186/s13007-019-0418-8)

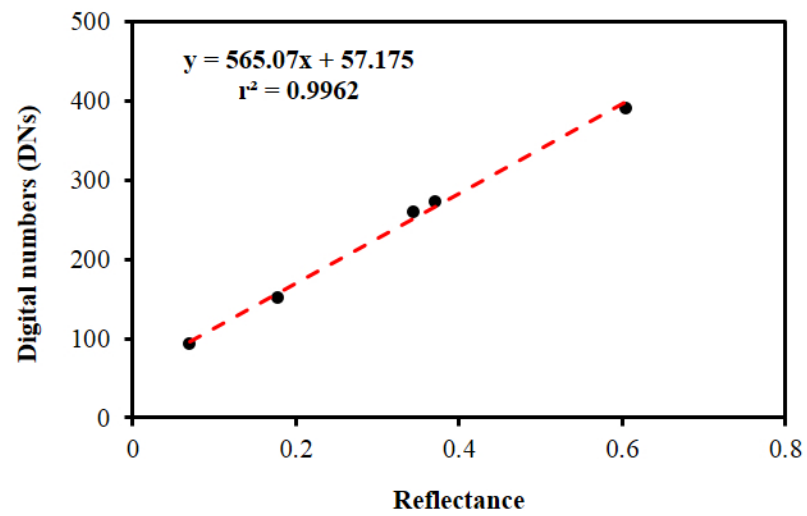

Fig. 2S Radiation calibration result at 796 nm

Supplement: Supplementary file 2 — Additional file 2. Radiation calibration result at 796 nm. [file 13007_2019_418_MOESM2_ESM.pdf]
